# Factors Associated With Willingness to Undergo Bilateral Tubal Ligation Among Women Attending Antenatal and Family Clinics of UNIOSUN Teaching Hospital, Osogbo, Nigeria

**DOI:** 10.7759/cureus.92507

**Published:** 2025-09-17

**Authors:** Adeniyi Fasanu, Kehinde Awodele, Sunday C Adeyemo, Daniel A Adekanle, Johnson O Komolafe, Godwin Oyewumi, Oluwafunmilayo A Fasanu, Olufemi E Abidoye, Samuel O Omopariola, Eniola D Olabode

**Affiliations:** 1 Obstetrics and Gynaecology, Osun State University, Osogbo, NGA; 2 Public Health Research, University of Wolverhampton, Wolverhampton, GBR; 3 Health and Biomedical Sciences, Institut Supérieur de Santé, Niamey, NER; 4 Public Health Pharmacy, Primary Health Care Development Board, Osogbo, NGA; 5 Obstetrics and Gynaecology, East Kent Hospitals NHS Trust, Ashford, GBR; 6 Obstetrics and Gynaecology, All Women's Care Fertility and Specialist Hospital (Shiloh), Osogbo, NGA; 7 Obstetrics and Gynaecology, Obafemi Awolowo University Teaching Hospitals Complex, Ile-Ife, NGA

**Keywords:** antenatal clinic, bilateral tubal ligation, fallopian tube block, family planning clinic, female reproductive health, female sterilization, permanent birth control, sub-saharan africa, tubectomy

## Abstract

Background: Bilateral tubal ligation (BTL) is a surgical and permanent form of contraception offered to women who have completed their family size or for the limitation of family size due to medical conditions. The practice of BTL is, however, limited in Nigeria and other sub-Saharan African countries, due to religious and sociocultural factors, as well as misunderstanding and fear of the procedure. Therefore, this study aims to compare the willingness to undergo BTL among women attending antenatal and family planning clinics in Osogbo.

Methods: This study was a descriptive comparative study carried out over a six-week period among women attending the antenatal and family planning clinics from December 2022 to January 2023. A minimum sample size of 123 was determined for each group. Data was obtained using a semi-structured self-administered questionnaire. Data analysis was done with IBM SPSS Statistics for Windows, version 22 (IBM Corp., Armonk, New York, United States). Chi-square test was used to determine potential statistically significant differences between the two groups. Binary logistic regression was used to predict willingness to undergo BTL. A p-value < 0.05 was considered statistically significant.

Results: A total of 120 questionnaires and 100 questionnaires were properly filled out and returned from the antenatal and family planning clinics, respectively. Overall, willingness to uptake BTL was higher among women attending the family planning clinic than among those attending the antenatal clinic. Logistic regression showed that age ≥36 years (adjusted OR (AOR) = 2.30, 95%CI: 1.28-4.12, p = 0.005), having ≥2 children (AOR = 2.92-4.76, p < 0.01), desiring more than five children with a partner (AOR = 3.92, p = 0.005), and an interval of more than five years since last delivery (AOR = 3.17, p = 0.003) significantly predicted willingness for BTL. Partner support was the strongest predictor (AOR = 4.25, 95% CI: 2.38-7.59, p < 0.001).

Conclusion: Willingness to undergo BTL is higher among older, high-parity women and strongly influenced by partner approval. Interventions to improve BTL acceptance should prioritize couple-focused counselling, engage men in reproductive health decisions, and integrate BTL education into antenatal services.

## Introduction

Bilateral tubal ligation (BTL) is a permanent form of birth control for women. It involves a surgery where the fallopian tubes are blocked or sealed so that pregnancy cannot happen. As an effective permanent contraceptive method, BTL (or occlusion/blockage/sterilization) has gained increased global awareness and public acceptance, alongside the long-acting reversible contraceptive (LARC) methods [1]. Despite this, the use of BTL is still low in many developing countries, including Nigeria.

The factors responsible for poor uptake of BTL are deeply entrenched in sociocultural and religious influence [2]. Educational status, spousal influence, productivity desire, anticipated stigma, and loss of self-esteem are some common factors documented in literature to affect uptake [3]. Other factors include the permanence of BTL, unexpected child loss, and anticipated side effects [4]. The cost of the surgery can also hinder some women from choosing it, especially when it is not free or subsidized [5]. On the other hand, women attending antenatal clinics (ANCs) and receiving counseling at family planning clinics (FPCs) can be encouraged to consider BTL [6].

While many studies in Nigeria have looked at general contraceptive use, few have focused on the factors that influence women’s willingness to choose permanent methods like BTL. This gap in knowledge is important, especially in Nigeria, where targeted education and support could help women make better family planning decisions. Understanding the factors associated with the willingness to uptake BTL is important for designing programs that address the specific needs and concerns of women in the community.

Therefore, this study aims to compare the willingness to undergo BTL among women attending ANCs and FPCs in Osogbo, Nigeria. The findings of this study will provide information that can guide policies and improve family planning service delivery, leading to better reproductive health outcomes for women in Osogbo and similar communities.

## Materials and methods

This was a descriptive comparative cross-sectional study conducted over six weeks, from December 2022 to January 2023, at the ANC and FPC of the Osun State University (UNIOSUN) Teaching Hospital, Osogbo, Nigeria. The study was approved by the Ethics and Research Committee of Osun State University Teaching Hospital (reference number: UTH/REC/2025/03/1168). Written informed consent was obtained from all participants before data collection. Confidentiality and anonymity were ensured by using codes instead of personal identifiers. Participants were informed of their right to withdraw from the study at any time without any consequences to their medical care.

Eligibility criteria

Inclusion Criteria

Women of reproductive age (15-49 years) attending the ANC or FPC of the UNIOSUN Teaching Hospital during the study period, who gave written or verbal informed consent to participate in the study, were included in the study.

Exclusion Criteria

Women who declined consent, those with severe illness that hindered participation, and those who had previously undergone BTL were excluded.

Sample size determination

The minimum sample size for each group was calculated using the formula:

$n = \frac{\left(z_{\alpha/2} + z_{\beta}\right)^2 \, \left[p_1 \left(1 - p_1\right) + p_2 \left(1 - p_2\right)\right]}{\left(p_1 - p_2\right)^2}$

where z_α/2_​ is the standard normal deviate at 95% confidence level (1.96), z_β_ corresponds to 80% power (0.84), p_1_​ = 0.171 [7] and p_2_​ = 0.389 [8], which are the estimated proportions of the outcome in the two groups. A minimum sample size of 123 was determined for each group.

Sampling technique

A systematic sampling technique was employed. For the ANC group, a list of 250 registered women during the study period was obtained from the records unit. The sampling interval was calculated as 250 ÷ 123 ≈ 2. The first respondent was selected at random, while subsequent respondents were chosen at every second interval until the required sample size was obtained. For the FPC group, a total of 120 women were registered during the study period. All eligible clients who gave consent were recruited to achieve the required sample size.

Data collection

Quantitative data were collected using a pre-tested, structured, self-administered questionnaire (see Appendices). The questionnaire assessed sociodemographic characteristics, family and reproductive characteristics, and willingness to undergo BTL. The questionnaire was in English and translated into the Yoruba language for those whose comprehension of the English language is inadequate.

Data analysis

Data were entered and analyzed using IBM SPSS Statistics for Windows, version 22 (IBM Corp., Armonk, New York, United States). Descriptive statistics (frequencies, percentages, means, and standard deviations) were generated. The Chi-square test was used to determine statistically significant associations between categorical variables. A p-value of <0.05 was considered statistically significant.

## Results

A total of 120 and 100 properly completed questionnaires were retrieved from the ANC and FPC, respectively.

Sociodemographic characteristics of respondents

The age of participants ranged from 18 to 45 years. The mean age of respondents from the ANC was 31.4 ± 6.2 years, while that of those in the FPC was significantly higher at 36.8 ± 7.1 years. When categorized into age groups, the largest proportion of ANC respondents (n=54, 45.0%) fell within the age group of 26-35 years, whereas the FPC was predominantly within the age group of 36-45 years (n=44, 44.0%). The differences observed in age distribution between the two groups were statistically significant (χ² = 20.45, t = -6.07, p < 0.001). Most respondents in both the APC (n=110, 91.7%) and FPC (n=94, 94.0%) groups were married. Across both groups, the majority of women had attained tertiary education (ANC: 64, 53.3%; FP: 56, 56.0%). Regarding employment, most respondents were self-employed (ANC: 56, 46.7%; FP: 52, 52.0%). Christianity was the most practiced religion among respondents (ANC: 74, 61.7%; FP: 58, 58.0%). These differences were not statistically significant (Table 1).

**Table 1:** Sociodemographic characteristics of respondents Data presented in frequency and percentage, except for age, which is given as mean ± SD χ² = chi-square, df = degree of freedom, p = p-value, *= statistically significant

Variables	Antenatal Clinic (n=120), n (%)	Family Planning (n=100), n (%)	Test Statistics	p-value
Age Group (years)				
18–25	28 (23.3%)	8 (8.0%)	χ² = 20.45	*p < 0.001
26–35	54 (45.0%)	30 (30.0%)		
36–45	30 (25.0%)	44 (44.0%)		
>45	8 (6.7%)	18 (18.0%)		
Age (years), mean ± SD	31.4 ± 6.2	36.8 ± 7.1	t = - 6.07	*p < 0.001
Marital Status				
Married	110 (91.7%)	94 (94.0%)	χ² = 0.41	p = 0.52
Single	10 (8.3%)	6 (6.0%)		
Level of Education				
Primary	14 (11.7%)	10 (10.0%)	χ² = 0.19	p = 0.91
Secondary	42 (35.0%)	34 (34.0%)		
Tertiary	64 (53.3%)	56 (56.0%)		
Employment Status				
Employed	40 (33.3%)	30 (30.0%)	χ² = 0.63	p = 0.73
Self-employed	56 (46.7%)	52 (52.0%)		
Unemployed	24 (20.0%)	18 (18.0%)		
Occupation				
Banking	6 (5.0%)	4 (4.0%)	χ² = 3.25	p = 0.78
Business	18 (15.0%)	22 (22.0%)		
Civil Service	16 (13.3%)	12 (12.0%)		
Health Worker	10 (8.3%)	6 (6.0%)		
Teacher	20 (16.7%)	14 (14.0%)		
Trading	30 (25.0%)	26 (26.0%)		
Vocational	12 (10.0%)	10 (10.0%)		
Others	8 (6.7%)	6 (6.0%)		
Religion				
Christianity	74 (61.7%)	58 (58.0%)	χ² = 0.29	p = 0.87
Islam	44 (36.7%)	40 (40.0%)		
Traditional	2 (1.6%)	2 (2.0%)		

Family characteristics of respondents

The majority of women reported living in a monogamous family structure, accounting for 82 (68.3%) in the ANC group and 70 (70.0%) in the FPC group. This was not statistically significant (p = 0.90). Parity distribution varied significantly between the two groups (χ² = 52.14, p < 0.001). Among the ANC respondents, the majority (46, 38.3%) had two to three children, while 30 (25.0%) had one child, 30 (25.0%) had four to five children, and 14 (11.7%) had no children. In contrast, most women in the FPC group (n=60, 60.0%) had four to five children, followed by 34 (34.0%) with two to three children, while none reported having no children. In terms of desired family size, 45.0% of ANC respondents and 34.0% of FPC respondents agreed with their partners on having two to three children. Similarly, 50 (41.7%) and 46 (46.0%) ANC and FPC respondents, respectively, opted for four to five children. The differences between the groups were statistically significant (χ² = 8.26, p = 0.041).

Income distribution showed that 35.0% of ANC and 28.0% of FPC respondents earned ₦50,000-100,000 monthly. Decision-making patterns regarding the choice of family planning method were similar across groups. In the ANC group, 74 (61.7%) reported joint decision-making with their partners, compared to 62 (62.0%) in the FPC group. However, these differences were not statistically significant.

Significant differences were observed in the time interval since the last delivery (χ² = 64.21, p < 0.001). Among ANC respondents, 48 (40.0%) reported one year since last delivery, followed by 36 (30.0%) with two to three years. In contrast, in the FPC group, most respondents (n=38, 38.0%) reported more than five years, followed by 30 (30.0%) with two to three years. Support for BTL varied slightly between groups. Among ANC respondents, 78 (65.0%) believed their partners would support the decision, compared to 76 (76.0%) in the FPC. Lack of support was reported by 42 (35.0%) and 24 (24.0%) in ANC and FPC groups, respectively. The difference was not statistically significant (Table 2).

**Table 2:** Family characteristics of respondents χ² = chi-square, df = degree of freedom, p = p-value, * = statistically significant

Variables	Antenatal Clinic (n=120), n (%)	Family Planning (n=100), n (%)	Statistics
Family Setting			
Monogamous	82 (68.3%)	70 (70.0%)	χ² = 0.21, df = 2, p = 0.90
Polygamous	32 (26.7%)	24 (24.0%)	
Cohabiting	6 (5.0%)	6 (6.0%)	
Number of children			
None	14 (11.7%)	0 (0.0%)	χ² = 52.14, df =3, *p < 0.001
1	30 (25.0%)	6 (6.0%)	
2–3	46 (38.3%)	34 (34.0%)	
4–5	30 (25.0%)	60 (60.0%)	
Agreed number of children with partner			
1	4 (3.3%)	2 (2.0%)	χ² = 8.26, df = 3, *p = 0.041
2–3	54 (45.0%)	34 (34.0%)	
4–5	50 (41.7%)	46 (46.0%)	
>5	12 (10.0%)	18 (18.0%)	
Monthly Family Income (₦)			
<50,000	28 (23.3%)	18 (18.0%)	χ² = 4.98, df = 3, p = 0.17
50,000–100,000	42 (35.0%)	28 (28.0%)	
100,001–200,000	34 (28.3%)	32 (32.0%)	
>200,000	16 (13.3%)	22 (22.0%)	
Who chooses the family planning method?			
Self	28 (23.3%)	16 (16.0%)	χ² = 2.84, df =2, p = 0.24
Partner/Spouse	18 (15.0%)	22 (22.0%)	
Both	74 (61.7%)	62 (62.0%)	
Interval from last delivery (years)			
1	48 (40.0%)	4 (4.0%)	χ² = 64.21, df = 3, *p < 0.001
2–3	36 (30.0%)	30 (30.0%)	
4–5	24 (20.0%)	28 (28.0%)	
>5	12 (10.0%)	38 (38.0%)	
Will your partner support your decision for BTL?			
No	42 (35.0%)	24 (24.0%)	χ² = 3.11, df =1, p = 0.078
Yes	78 (65.0%)	76 (76.0%)	

Willingness to undergo BTL

**Figure 1:**
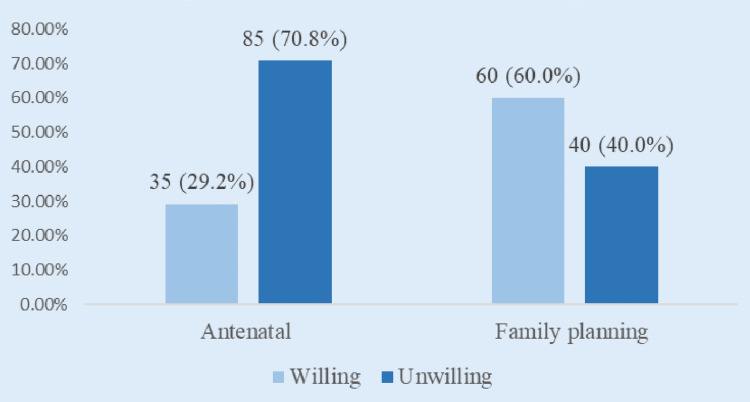
Willingness to uptake bilateral tubal ligation among respondents from the antenatal clinic and family planning clinic Data is presented in frequency and percentage

Among ANC respondents, 35 (29.2%) indicated willingness to undergo BTL, while 85 (70.8%) were unwilling. In the FPC group, 60 (60.0%) were willing, while 40 (40.0%) were unwilling (Figure 1). This difference was statistically significant using the chi-square test (ꭓ2 = 14.133, p = 0.002, df =1) (Figure 1).

Predictors of willingness to undergo BTL

Compared to women aged 18-25 years, those in the age group of 36-45 years had more than twice the odds of willingness to uptake BTL (adjusted OR (AOR) = 2.30, 95%CI: 1.28-4.12, p = 0.005), while women above 45 years had nearly four times higher odds (AOR = 3.85, 95%CI: 1.72-8.61, p < 0.001). The age group of 26-35 years showed no significant association (AOR = 1.42, 95%CI: 0.82-2.45, p = 0.21).

Parity was a strong predictor of BTL uptake. Compared with women who had no children, those with two to three children (AOR = 2.92, 95%CI: 1.41-6.04, p = 0.004) and four to five children (AOR = 4.76, 95% CI: 2.15-10.54, p < 0.001) were significantly more likely to accept BTL. Having one child was not associated with increased odds (AOR = 1.64, 95% CI: 0.74-3.62, p = 0.23).

Compared to women who agreed on having only one child, those who desired more than five children with their partners were almost four times more likely to accept BTL (AOR = 3.92, 95%CI: 1.51-10.18, p = 0.005). Agreement on two to three children or four to five children did not significantly influence BTL willingness (p > 0.05).

A longer interval since the last delivery was associated with an increased likelihood of BTL acceptance. Specifically, women with more than five years since last delivery were over three times more likely to accept BTL compared with those with only one year (AOR = 3.17, 95%CI: 1.48-6.81, p = 0.003). The associations for two to three years and four to five years were not statistically significant.

Partner’s support emerged as the strongest predictor. Women who reported partner support for BTL were over four times more likely to accept the procedure compared with those without support (AOR = 4.25, 95%CI: 2.38-7.59, p < 0.001) (Table 3).

**Table 3:** Factors associated with willingness to uptake bilateral tubal ligation BTL: bilateral tubal ligation; FP: family planning; ₦: Naira

Predictors	Adjusted Odds Ratio (AOR)	95% Confidence Interval (CI)	p-value
Age group (Ref: 18–25 years)			
26–35 years	1.42	0.82 – 2.45	0.21
36–45 years	2.30	1.28 – 4.12	0.005*
>45 years	3.85	1.72 – 8.61	<0.001*
Number of children (Ref: None)			
1	1.64	0.74 – 3.62	0.23
2–3	2.92	1.41 – 6.04	0.004*
4–5	4.76	2.15 – 10.54	<0.001*
Agreed number of children with partner (Ref: 1)			
2–3	1.38	0.56 – 3.41	0.47
4–5	2.11	0.88 – 5.08	0.09
>5	3.92	1.51 – 10.18	0.005*
Monthly family income			
50,000–100,000	1.12	0.60 – 2.09	0.72
100,001–200,000	1.45	0.74 – 2.82	0.28
>200,000	2.05	0.94 – 4.49	0.07
Who chooses FP method (Ref: Self)			
Partner/Spouse	0.88	0.44 – 1.74	0.72
Both	1.34	0.73 – 2.44	0.34
Interval from last delivery (Ref: 1 year)			
2–3 years	1.22	0.64 – 2.35	0.54
4–5 years	1.86	0.93 – 3.73	0.08
>5 years	3.17	1.48 – 6.81	0.003*
Partner support for BTL (Ref: No)	4.25	2.38 – 7.59	<0.001*

## Discussion

This study examined the willingness to uptake BTL and its associated factors among women attending ANCs and FPCs in a tertiary hospital in Southwestern Nigeria. It found that women at FPCs were usually older and more likely to have four or more children than women at APCs. This supports earlier research in Nigeria and other developing countries, which showed that older women and those with larger families are more open to permanent contraception [3,7]. Older women often feel their families are complete, which explains why they are more willing to accept BTL. In contrast, younger women, especially those under 30 years of age with one or no children, preferred temporary methods and were less interested in sterilization.

This study revealed significant differences in the willingness to undergo BTL between the two groups. Among ANC respondents, only 35 (29.2%) were willing to undergo BTL, while in the FPC group, 60 (60.0%) indicated willingness. Higher willingness in the FPC group was expected, since women who were visiting FPCs are more broadly exposed and educated on various options of family planning methods, including BTL. Education on contraception is not often prioritized in ANCs, as the focus seems to be on ensuring safe delivery outcomes for mother and baby. This is a major cause for concern, as it’s been found that women who were adequately counseled in time during ANC visits about BTL would usually have enough time to think it through, and make informed decisions, with increased uptake as a result, compared to women who were not. This finding aligns with the study of Balogun et al., who reported that access to contraceptive counseling improves women’s acceptance of long-term and permanent family planning methods [6]. Similarly, Ewerling et al. noted that women with past contraceptive use and more knowledge about their reproductive health were more open to BTL [8].

The number of children was strongly associated with willingness for BTL, such that women with four or more children were almost five times more likely to accept BTL than women with no children. Similar results were seen in studies from northern Nigeria [9] and Kenya [10], showing that having more children increases the likelihood of accepting tubal ligation. This suggests that parity is an important factor for permanent contraception across sub-Saharan Africa. The study also showed that women whose last birth was more than five years ago were more willing to consider BTL. This may indicate that women are ready to stop having children after a long break, a finding also seen in a previous study [3].

Partner support was the strongest factor in this study. Women whose partners approved of BTL were over four times more likely to want the procedure than women without partner support. This shows how much influence men have on family planning decisions in Nigeria, where cultural traditions often give husbands strong control [4]. Similar results have been reported in Ghana, where spousal approval strongly affected contraceptive use [11]. Thus, interventions aimed at improving BTL acceptance should actively engage male partners through couple-focused counselling.

Interestingly, household income and educational attainment did not significantly influence willingness for BTL. This finding contrasts with studies from high-income countries where socioeconomic status is often a determinant of sterilization decisions [2]. However, in the Nigerian context, cultural and reproductive norms may play a more dominant role than economic factors in shaping decisions about permanent contraception.

Strengths and limitations

Strengths include the comparison of women visiting ANCs and FPCs and a multivariable model that isolates independent predictors. Limitations include the clinic-based sample and cross-sectional design, which limit the generalizability of the study. Also, self-reported measures may be influenced by social desirability. Additionally, the non-uniform sample selection in the ANC and FPC might have introduced a certain degree of selection and cognitive bias. 

Recommendations

Strengthen Counselling Services

ANCs and FPCs should provide comprehensive counselling on permanent contraceptive methods, emphasizing the safety, effectiveness, and long-term benefits of BTL.

Promote Male Partner Involvement

Health facilities should develop couple-focused counselling sessions and outreach programmes to actively involve male partners, as their approval significantly increases women’s willingness to uptake BTL.

Target High-Parity Women

Special attention should be given to women with four or more children and those with long intervals since last delivery, as they are more likely to benefit from permanent contraception.

Integrate BTL Education in Antenatal Care

Early education on BTL during antenatal visits can improve awareness and dispel misconceptions, particularly among younger women who may consider permanent contraception in the future.

Community Engagement

Public health campaigns should leverage community and religious leaders to normalize discussions around permanent contraception and reduce cultural barriers to BTL acceptance.

Further Research

Longitudinal and qualitative studies are needed to explore the perceptions, barriers, and long-term satisfaction of women and couples who accept BTL in Nigeria.

## Conclusions

This study identified that willingness to accept BTL is significantly associated with older age, higher parity, a longer interval since the last delivery, and, most importantly, partner support. These findings highlight the critical role of spousal involvement in reproductive health decisions within this cultural context. However, the authors acknowledge that willigness may not be equal to uptake.

Understanding these factors is vital for designing effective reproductive health programs. By implementing targeted, couple-centred interventions and integrating early education on permanent methods into routine care, healthcare providers can better support women in making informed choices about their reproductive futures, ultimately improving family planning outcomes.

## Figures and Tables

**Figure 2:**
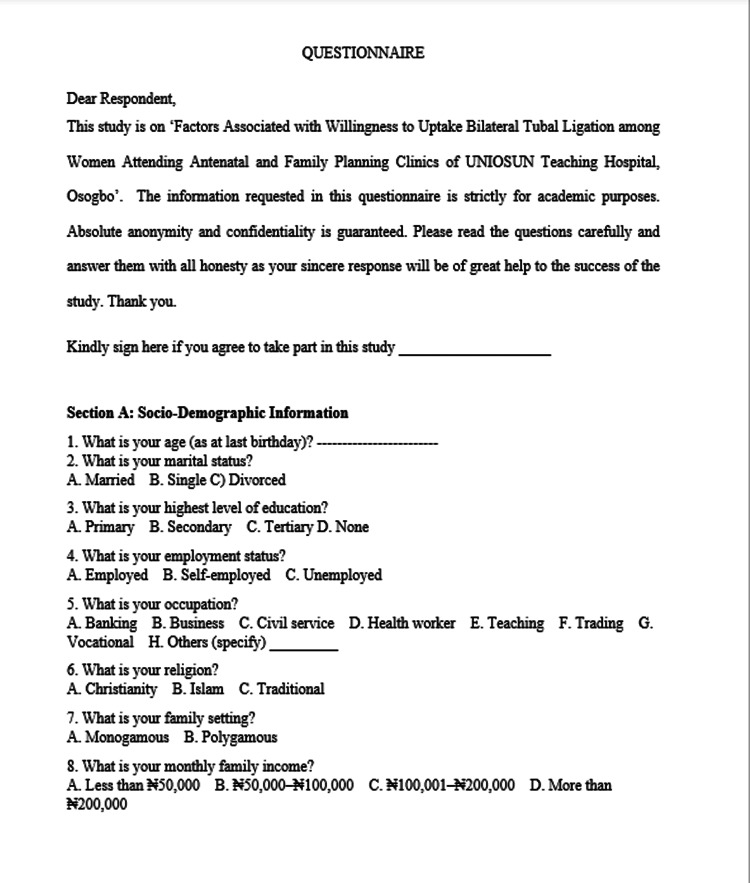
Study questionnaire page 1

**Figure 3:**
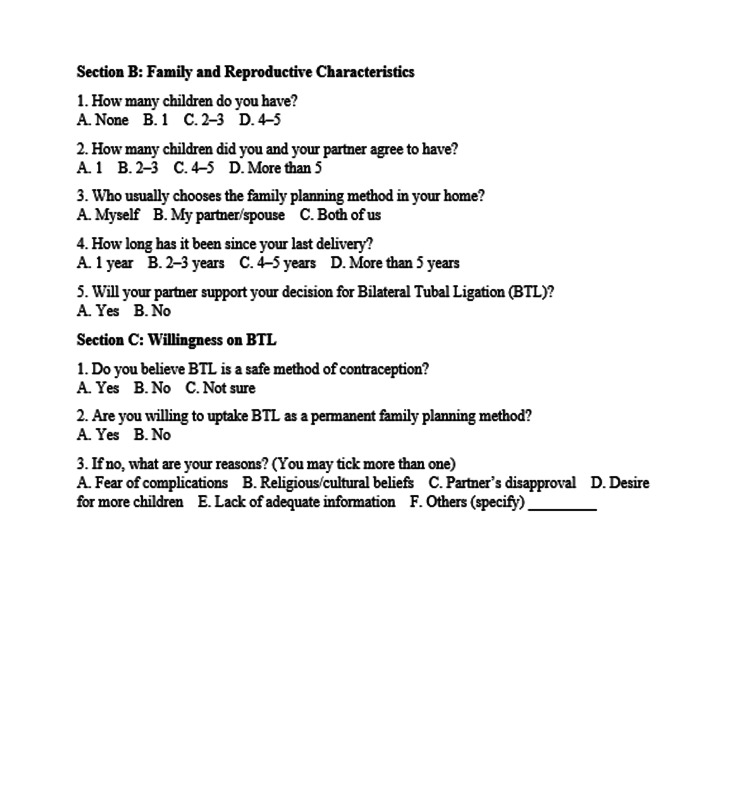
Study questionnaire page 2
